# Consolidated Newborn Bloodspot Screening Efforts in Developing Countries in the Asia Pacific—2024

**DOI:** 10.3390/ijns11010002

**Published:** 2024-12-30

**Authors:** Bradford L. Therrell, Carmencita D. Padilla, Michelle E. Abadingo, Shree Prasad Adhikari, Thuza Aung, Thet Thet Aye, Sanjoy Kumer Dey, Muhammad Faizi, Erdenetuya Ganbaatar, Tran Thi Huong Giang, Hoang Thu Hang, Rathmony Heng, Seema Kapoor, Khurelbaatar Nyamdavaa, Prajwal Paudel, Kimyi Phou, Aman B. Pulungan, Chittaphone Sayyavong, Salimah R. Walani, Tariq Zafar

**Affiliations:** 1 Department of Pediatrics, University of Texas Health Science Center San Antonio, San Antonio, TX 78229, USA; 2 National Newborn Screening and Global Resource Center, Austin, TX 78759, USA; 3 Department of Pediatrics, College of Medicine, University of the Philippines Manila, Manila 1000, Philippines; cdpadilla@up.edu.ph (C.D.P.); meabadingo@up.edu.ph (M.E.A.); 4 Newborn Screening Reference Center, National Institutes of Health, University of the Philippines Manila, Manila 1000, Philippines; 5 Paropakar Maternity and Women’s Hospital, Thapathali, Kathmandu 44600, Nepal; shreeprasad2063@gmail.com (S.P.A.); prajwal.paudel999@gmail.com (P.P.); 6 CLL Health, 16 Shin Saw Pu Street, Ahlone, Yangon 11211, Myanmar; thuzaaung.paed@gmail.com; 7 Nay Pyi Taw Children Hospital, Oketarathiri 15015, Myanmar; dr.thet.thet.aye@gmail.com; 8 Department of Neonatology, Bangabandhu Sheikh Mujib Medical University, Shahbag, Dhaka 1000, Bangladesh; skdey19@gmail.com; 9 Department of Child Health Medical Faculty, Universitas Airlangga, Dr. Soetomo Hospital, Jl. Prof. Dr. Moestopo, Surabaya 60286, Indonesia; muhammad.faizi@fk.unair.ac.id; 10 Department of Pediatrics, School of Medicine, Mongolian National University of Medical Sciences, Zorig Street, Ulaanbaatar 14210, Mongolia; erdenetuya@mnums.edu.mn; 11 Department of Population Structure and Quality, Vietnam Population Authority (VNPA), 8 Alley, Ton That Thuyet Street, My Dinh 2 Ward, Nam Tu Liem District, Hanoi 12014, Vietnam; tranhuonggiang.bg@gmail.com; 12 Hanoi Population Branch, Department of Health, Hanoi 12014, Vietnam; hanghoangthu2109@gmail.com; 13 Neonatology Department, Calmette Hospital, Phnom Penh 12201, Cambodia; ilovecambodia3000@yahoo.com; 14 Division of Genetics, Genetic & Metabolic Laboratory, Department of Pediatrics, Lok Nayak Hospital & Maulana Azad Medical College, New Delhi 110002, India; drseemakapoor@gmail.com; 15 Development Projects and International Affairs, Mongolian National University of Medical Sciences, Zorig Street, Ulaanbaatar 14210, Mongolia; khurelbaatar.n@mnums.edu.mn; 16 Calmette Hospital, Phnom Penh 12201, Cambodia; kimyiphou@yahoo.fr; 17 Department of Child Health, Faculty of Medicine, Universitas Indonesia, Jakarta 10430, Indonesia; amanpulungan@mac.com; 18 Mother and Newborn Hospital, Ministry of Health, Vientiane 0100, Laos; chit_sv@yahoo.com; 19 School of Nursing and Midwifery, The Aga Khan University, Stadium Road, Karachi 74800, Pakistan; salimah.walani@aku.edu; 20 Independent Researcher, 307-N, Street 113, Phase 1, DHA, Lahore 54792, Pakistan; drtzafar@hotmail.com

**Keywords:** newborn screening, Asia Pacific, bloodspot, NBS, DBS, IAEA, APSHG

## Abstract

Approximately half of all births globally occur in the Asia Pacific Region. Concerted efforts to support local activities aimed at developing national newborn screening (NBS) have been ongoing for almost 30 years, first by the International Atomic Energy Agency (IAEA) and then through volunteer efforts. Sustainable newborn bloodspot screening (NBS) continues to be initiated and develop in many of the countries with developing economies in the region. Since the discontinuation of IAEA funding in 2007, a working group of the Asia Pacific Society of Human Genetics (APSHG) consisting of interested representatives from countries in the region with less than 50% NBS coverage has participated in periodic workshops to exchange information, set goals, and provide peer support. Facilitated by international NBS experts, interested corporate sponsors, and the APSHG, the 7th workshop of representatives from 10 East Asian countries with developing NBS systems was recently held in Kathmandu, Nepal. This report summarizes the NBS activities in these countries and describes the continuing efforts to move NBS ahead in the region.

## 1. Introduction

Newborn bloodspot screening (NBS) is a well-recognized public health prevention program that exists in most high-income (HIC) and many upper middle-income (UMIC) countries with more developed economies and many low- and low middle-income countries (LICs and LMICs) with developing or depressed economies [1,2]. The use of bloodspot specimens to screen newborns for phenylketonuria (PKU) was pioneered by Guthrie, who first reported their use in 1959 [3]. He reported an analysis of the first screening specimens transported to his laboratory from nearby hospitals beginning in 1962 as “the beginning of NBS”, with formal introduction of NBS as part of the state public health efforts occurring a year later [4]. While most US states had established NBS programs by the end of the 1960s, bloodspot screening programs had only begun in a few locations in the Asia Pacific region—Australia, Japan, and New Zealand [5]. Over the years, other NBS programs have been initiated in the region, and reviews/summaries of their progress have been published [1,5,6].

**Figure 1:**
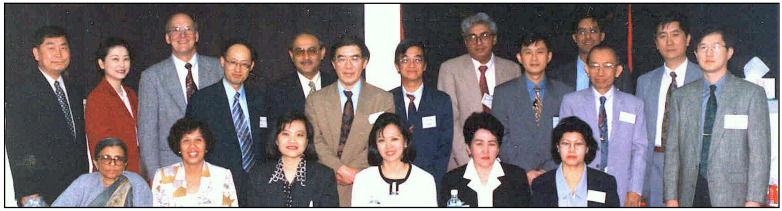
Representatives at first regional meeting of Asian countries, 17–21 May 1999. Seated L to R: Fauzia Moslem (Bangladesh), Nguyen Thi Hoan (Vietnam), Wiyada Charoensiriwatana (Thailand), Carmencita Padilla (the Philippines), Oyun Nanzad (Mongolia), Diet Sadiah Rustama (Indonesia—Bandung). Standing L to R: Unidentified Observer, Hye-Ran Yoon (South Korea), Bradford Therrell (USA), Dong Hwan Lee (South Korea), Reyad Kamel (IAEA), Soo Lin Ch’ng (IAEA), San Aye (Myanmar), Shahid Kamal (Pakistan), Shi Lixin (China), Amar Singh (Malaysia), Frans Sardi Satyawirawan (Indonesia—Jakarta), Gao Shuo (China), Unidentified Observer.

To assist with developing NBS systems, the International Atomic Energy Agency (IAEA) Technical Cooperation Program reportedly spent an estimated 6.7 million USD on 29 Member Countries from 1995 through 2007 to encourage NBS for congenital hypothyroidism (CH) [7]. IAEA professional education efforts included periodic regional workshops/seminars for NBS project coordinators with internationally recognized content experts as faculty in a “train the trainer” format. In Asia, IAEA NBS collaborative workshops began in 1999 with representatives from 11 East Asian member countries—Bangladesh, China, Indonesia, Pakistan, the Philippines, Malaysia, Mongolia, Myanmar, South Korea, Thailand, and Vietnam (see Figure 1), eventually expanding to include 4 additional IAEA project partners from West Asia—Iran, Iraq, Sri Lanka, and the United Arab Emirates.

**Figure 2:**
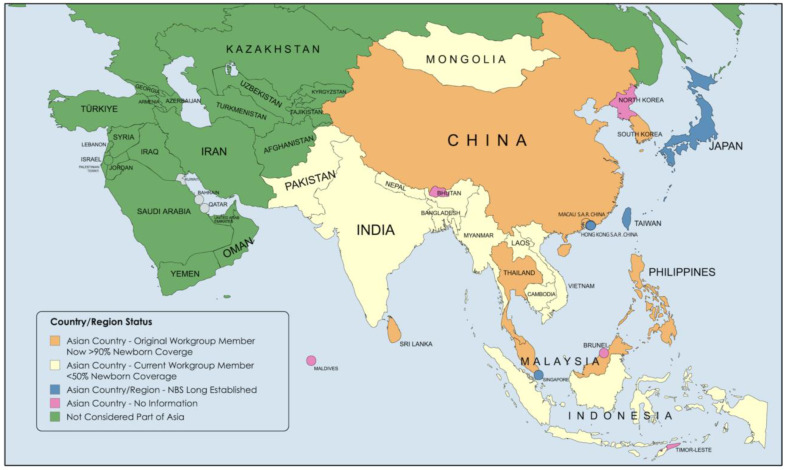
Map showing Asian countries previously and currently part of the Asia Pacific Working Group on Newborn Screening.

This information sharing and peer support approach was successful in reaching a large portion of the personnel needed for successful training and NBS implementation. A handbook for implementing NBS for congenital hypothyroidism (CH) was published in 2005 by the IAEA [8]. When IAEA funding ceased in 2007, the East Asia collaborators agreed to continue periodic meetings in a similar format (usually 3–4-day meetings), sharing information on the progress of NBS implementation, short- and long-term goal setting, and discussions of other subjects of interest to participants, with presentations and discussions from recognized international experts. Group participation was limited to countries with less than 50% newborn coverage and was open to all countries in the region meeting this criterion (see Figure 2).

The group was granted official affiliation as a working group of the Asia Pacific Society for Human Genetics (APSHG) in 2010. NBS is, and has been, a significant focus of the Asia Pacific Conferences of Human Genetics (sponsored by the APSHG), which have included plenary topics and parallel symposia on NBS since their beginning. Two of us, C.D.P. and B.L.T., both life members of APSHG, assumed the role of coordinators, soliciting funding and planning/organizing the workshops. Representatives from eleven countries attended the first group meeting in Cebu, the Philippines, held as a satellite meeting of the APSHG. Countries represented included Bangladesh, China, India, Indonesia, Palau, Pakistan, the Philippines, Mongolia, Myanmar, Sri Lanka, and Vietnam, along with observers and expert consultants from the U.S. Centers for Disease Control and Prevention (CDC), the U.S. National Institutes of Health (NIH), the U.S. National Newborn Screening and Global Resource Center (NNSGRC), and the International Society for Neonatal Screening (ISNS) [9].

**Figure 3:**
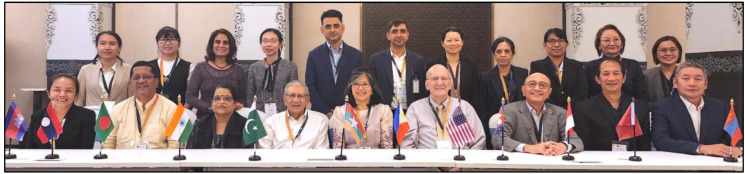
Participants in the 7th Workshop on Consolidating Newborn Screening Efforts in the Asia Pacific, Kathmandu, Nepal, 2–5 September 2024. Seated L to R: Chittaphone Sayyavong (Laos), Sanjoy Kumer Dey (Bangladesh), Seema Kapoor (India), Tariq Zafar (Pakistan), Carmencita Padilla (the Philippines), Bradford Therrell (USA), Aman Pulungan (Indonesia), Muhammad Faizi (Indonesia), and Khurelbaatar Nyamdavaa (Mongolia). Standing L to R: Rathmony Heng (Cambodia), Hoang Thu Hang (Vietnam), Salimah Walani (Pakistan), Kimyi Phou (Cambodia), Prajwal Paudel (Nepal), Shree Prasad Adhikari (Nepal), Tran Thi Huong Giang (Vietnam), Thuzar Aung (Myanmar), Thet Thet Aye (Myanmar), Erdenetuya Ganbaatar (Mongolia), and Michelle Abadingo (the Philippines).

While Malaysia, South Korea, and Thailand were part of the original East Asia IAEA project meetings (1999–2007), their successful expansion to full national coverage resulted in all three not being part of the group that continued after IAEA support ended in 2007. Since 2008, seven meetings focused on consolidating NBS activities in the Asia Pacific have been held, with the seventh in Kathmandu, Nepal, having recently been concluded (see Figure 3). Over the years, screening coverage in China, Sri Lanka, and the Philippines has expanded beyond the 50%, the threshold for working group membership, and representatives from these countries have been included in workshops only on an occasional basis as subject matter experts.

Each meeting has had a slightly different focus, but all have emphasized sharing information and experiences aimed at establishing national, population-wide, and sustainable NBS systems. As output from most meetings, a consensus declaration was created for sharing with the participating country’s health ministries to demonstrate unity of purpose within the region and to provide updated information on NBS. The intent of each declaration has been to gain confidence in, and support for, NBS as an essential health prevention strategy from each health minister. Here, we report the results of the 7th Workshop of the Asia Pacific Society of Human Genetics Society (APSHG) Working Group on Consolidating Newborn Bloodspot Screening Efforts in the Asia Pacific Region.

## 2. Methods

The 7th Workshop on Consolidating Newborn Bloodspot Screening Efforts in the Asia Pacific Region was held in Kathmandu, Nepal, on 2–5 September 2024. Organization of the meeting was a collaborative effort between the Philippine Newborn Screening Reference Center (NSRC, Manila, the Philippines), the US National Newborn Screening and Global Resource Center (NNSGRC, Austin, TX, USA), and the APSHG. The logistics were coordinated by the NSRC. Invited participants included country representatives who had previously participated in meetings of this working group from India, Pakistan, and Mongolia, along with newly committed/recruited representatives from Cambodia, Indonesia, Laos, Myanmar, Nepal, Pakistan, and Vietnam. Subject matter expertise was provided by the NNSGRC, having provided external NBS expertise since working group implementation by IAEA in 1999, and the NSRC, the Philippines having previously been a working group member and now with over 95% newborn population coverage (exceeding the 50% threshold for working group membership). A limited number of observers with an interest in achieving successful NBS services globally were present, primarily in a supportive role (NSRC staff and industry representatives seeking to assist in NBS implementation).

As part of the invitation process, participants were asked to commit to a work ethic focused on making NBS sustainable in their respective environments. They were also asked to review local, regional, and national NBS activities that might impact their NBS implementation/expansion efforts and to prepare a brief (1–2 pages; 3–5 slides) presentation for the group. Presentations were expected to describe national demographics, the local/national status of NBS, and obstacles to be overcome in creating a sustainable, national NBS system. Preparation of a second presentation defining future goals and actions to be accomplished were developed in consultation with other country participants with more experience. This second presentation also considered “lessons learned” from other country reports, expert presentations, and participant discussions.

## 3. Results

The workshop agenda was organized to allow participants from each country to share/update information on the status of NBS in their country and to set 1-, 2-, and 3-year goals for continued national NBS implementation. Participants from countries with ongoing NBS programs presented information on expansion progress in their country, while those with little or no formal NBS program discussed implementation plans. Each participant reported on national demography, NBS status, and obstacles to implementation and/or sustainability. Additionally, possible actions to overcome the implementation/expansion challenges were identified and briefly discussed. Comprehensive quality assurance of the NBS system was discussed using the USA NBS Performance Evaluation and Assessment Scheme (PEAS) [10] as the “gold standard” model and creation/use of the Philippine PEAS (PPEAS) as an example of PEAS development and use in a LMIC environment [11,12]. While some presenters also reported on expanded screening for newborn hearing loss (NHS) and critical congenital heart disease (CCHD), these activities were secondary to the purpose of our meeting and have not been emphasized here. Rather, the interested reader is referred to two reviews on international activities in these study areas [13,14].

Based on participant reports and literature review, brief summary reports outlining the history and current status for each participating country follow:

### 3.1. Bangladesh

Bangladesh, officially the People’s Republic of Bangladesh, is one of the most densely populated countries in the world, bordered by India on the north, east, and west and Myanmar to the southeast (see Figure 2). The southern coastline is along the Bay of Bengal, with mountains to the north. The population of approximately 175 million makes it the eight largest population, with almost three million births annually (ninth globally). Bangladesh is ethnically and culturally homogeneous, with Bengalis making up 99% of the population. The country is divided into 8 administrative divisions subdivided into 64 districts (zila) and 495 subdistricts (upazila), and the government operates as a unitary parliamentary system.

Pilot NBS for CH began in 1999 as part of a regional project of the IAEA through the Bangladesh Atomic Energy Commission (BAEC). Initial program support included equipment, filter paper collection cards, reagents, training and expert services. Between 1999 and 2006, 31,802 newborns were screened using cord blood absorbed onto filter paper in ten hospitals, primarily in Dhaka, and 16 newborns were diagnosed with hypothyroidism (1:1998) [15]. A second BAEC NBS project from 2006 through 2011 screened 220,000 newborns and found 96 cases of CH (1:2292). In 2009, during this project, the Ministry of Health and Welfare published its National Neonatal Health Strategy and Guidelines for Bangladesh, which noted in its “Guidelines for Interventions After Delivery” at the home and community level that “All neonates should be screened for congenital hypothyroidism …” [16].

In September 2018, the BAEC launched a new project in collaboration with the Ministry of Science and Technology (MOST): “Screening of Congenital Hypothyroidism in newborn Babies (Phase-2)”, with the intent to “decrease the child mortality rate by screening newborns at a very early age and provide treatment accordingly to achieve better demographic data of CH for Bangladesh”. In this project, 261,550 newborns from 669 hospitals and clinics covering 51 districts of Bangladesh were screened using heelstick blood, and 123 cases of CH were diagnosed (1:2126). The study authors proposed a government-run NBS program and a national policy to combat congenital diseases [17]. The incidence of CH in Bangladesh appears constant (~1:2100) and slightly higher than the global incidence of CH based on these three studies.

There has been a growing interest in NBS for conditions other than CH. A collaborative study between a research group in Ontario, Canada, and a group from Matlab, Bangladesh, in 2017/2018 described the frequency and nature of screen positive results for congenital, treatable conditions in a cohort of Bangladeshi infants. Particular emphasis was placed on screen positive cases of conditions where treatments were accessible and cost-efficient, using thresholds adjusted to increase certainty of the screen positive status. Conditions of interest in this regard included CH, medium-chain acyl co-A dehydrogenase deficiency (MCADD), and sickle cell diseases (SCDs). Screening 1661 newborns using a combination of heel stick and cord blood specimens dried onto filter paper, and comparing to Canadian screening thresholds, led to diagnoses of 61 cases of various disorders, with CH the most prevalent (29 cases). Early infant hospital discharge was identified as a screening challenge. In addition to the information on screening feasibility and disease prevalence, the authors encouraged continuation of the ongoing NBS efforts for CH [18].

A 2019 report described initial experiences using liquid chromatography tandem mass spectrometry (LC-MS/MS) to screen newborns for inborn metabolic disorders (IMDs) in Bangladesh. In addition to establishing the screening method, analysis of 273 specimens from patients suspected as having an IMD clinically yielded seven true IMD cases, three with PKU and one each with isovaleric acidemia (IVA), methylmalonic acidemia (MMA), carnitine uptake deficiency (CUD), and citrullinemia type-II (CIT-II). These results confirmed the feasibility of LC-MS/MS screening in Bangladesh and suggested conditions for consideration in a national NBS effort [19]. A 2022 report reviewed the current status of NBS for PKU in Bangladesh. Focus was on patient management and lifelong treatment, and the author suggested development of a government plan and national PKU screening policy [20].

A recent qualitative study assessed the current NBS practices in Bangladesh, focusing on the perspectives of healthcare professionals and identifying potential implications for enhancing infant health. The NBS challenges identified included limited awareness among healthcare professionals, inadequate infrastructure and resources, and absence of a comprehensive national NBS program. Financial constraints, cultural beliefs, and social barriers contributed to low NBS uptake by families. Lack of knowledge, attitudes, and access to NBS among health workers were identified as challenges. Action is needed to address medical education, clinical care, health policy, and information system’s needs. The authors suggest streamlined access to screening results, low-literacy information for parents, and clear communication guidelines for healthcare providers to address the challenges, noting that “community knowledge and mobilization are crucial for successful implementation”. The presence of a national diagnosed case database was also identified as a pressing need. Incorporating the perspectives of healthcare professionals and policymakers in developing targeted interventions was suggested to enhance NBS and improve infant health outcomes [21].

### 3.2. Cambodia

Cambodia, officially the Kingdom of Cambodia, is bordered by Thailand to the north and west, Laos to the northeast, Vietnam to the east and southeast, and shares 275 miles of coastline with the Gulf of Thailand (see Figure 2). The population is approximately 17 million, with ~320,000 births annually. There is a rainy season (May to October) and a dry season (November to April). There is a low-lying central plain surrounded by low mountains and uplands, with almost half of the country covered by forests. The Cambodian government is a constitutional monarchy with a bicameral parliament, prime minister (head of government), and king (head of state).

There is a general lack of awareness about NBS in Cambodia, and medical specialists are sparse. Since 2013, Cambodia has participated as a member of the Asia Pacific Working Group on NBS, and NBS for CH and glucose-6-phosphate dehydrogenase (G6PDD) has been offered in Calmette Hospital in Phnom based on a cost–benefit balance. While data on CH nationally are scarce, the reported prevalence of CH for the period 2013–2021 in Calmette Hospital is 1:2115. To date, the need for NBS in Cambodia is yet to be recognized nationally by health policymakers. Currently, there are no guidelines nor recommendations, and public sector NBS outside of Calmette Hospital does not exist. There is limited NBS in some private hospitals offered on a case-by-case basis and dependent on the type of delivery package insurance. Costs vary depending on the type of delivery and the NBS tests chosen by the parents [22].

Data continue to be collected in order to refine national information on disease prevalence for both CH and G6PDD. Table 1 provides data for the period 2013–2023, during which time, 123,493 newborns were screened: 59 cases of CH (1:2093) and 8527 cases of G6PDD (1:14.5; 6.9%). In Calmette Hospital, a NBS working group has been established, along with a NBS reference center for follow-up and management, a family education team, and a laboratory quality control system. NBS data are being assembled for presentation to the national congress and to professional society stakeholders, and these data will be published in the local university journal. A fee for NBS is being developed as part of government policies. The expansion of NBS to other hospitals is anticipated as word spreads regarding the program’s success at Calmette Hospital.

It is important to note that, once the infrastructure for NBS has been established, the program is more easily expanded to include other appropriate conditions, usually based on prevalence, testing and treatment availability, and cost–benefit balance. NBS for congenital adrenal hyperplasia, primarily due to 21-hydroxylase deficiency (CAH), is likely to fall into this category in the near future. A recent bi-national study of asymptomatic coronary heart disease among 3–18 year olds in China and Cambodia found a significant number of affected Cambodian children who might have benefited from NBS for CCHD [23]. Because NBS is not yet recognized nationally, it will be important to not only raise awareness of screening opportunities but also to acknowledge the difficulties of lifelong treatment in a low-resource country. One key to successful NBS and resultant pediatric case management is parental involvement, often influenced by education, culture, environment, and economics. The lack of specialty medical providers in Cambodia may make telemedicine a useful follow-up tool for NBS, affording parents the option to receive medical advice without the hardships related to time and travel, although limited digital literacy among the population presents another challenge.

### 3.3. India

India, officially the Republic of India, has a population of 1.44 billion, 17.78% of the global population. It has about 27 million births annually, one-fifth of the global births, and one-third of the neonatal deaths globally. It is the largest country by population, the seventh largest country by area, and has 4700 miles of coastline (see Figure 2). India is a federal union of 28 states and 8 union territories, with all states and three territories having elected legislatures. The other five union territories are directly ruled by the central government through appointed administrators. There is a bicameral parliament, an elected president (ceremonial head of state elected through electoral college—5-year term), and an appointed prime minister (head of government).

A significant decline in infant mortality rates in recent years has emphasized the potential value of establishing and adapting a sustainable NBS system to continue this trend. NBS becomes increasingly important as the infant mortality rate (IMR) approaches single-digits and NBS rises in public health importance as a prevention program. In addition to aligning with the 1999 World Health Organization’s (WHO’s) primary healthcare approaches for the prevention and control of congenital and genetic disorders, which included “neonatal screening using clinical and biochemical methods” [24], a comprehensive NBS program could significantly contribute to achieving the goals outlined in the 2014 India Newborn Action Plan (INAP), particularly the target of reducing neonatal mortality to single-digit figures by 2030 [25]. Despite reduced IMR in several Indian states (i.e., Goa, Kerala, Manipur, and Puducherry), there remains no consensus on the national implementation of NBS. There is, however, increasing realization that a reactive approach to medical issues instead of proactive prediction and prevention is economically wasteful. Consideration needs to be paid to the family’s social and emotional burdens and to the loss of a productive member of society.

Because of the large geographic and population sizes of India, it is likely that NBS will be implemented first on a state level, and pilots have trended in that direction. To that end, and noting a critical lack of national and state data on the incidence of screenable conditions, the Science and Engineering Research Board (SERB) of the Department of Science and Technology of the Government of India recently funded the first ever feasibility study for five NBS conditions in Delhi State, CH, CAH, BIO, GAL, and G6PDD and inborn metabolic disorders (IMDs) detectable by tandem mass spectrometry (MS/MS). A report on this study emphasizing and listing the issues encountered in translating a public health NBS program into routine neonatal healthcare and the concurrent requirement of an operational infrastructure has recently been published [26]. A recent report has also discussed the importance of high standards of program quality control and prompt diagnosis [27].

Historically, NBS efforts in India date to early studies of thyrotropin (TSH) levels in newborns in a Bombay hospital in 1987 [28] and aminoacidopathies in Bangalore [29]. There have been many NBS pilots across India over the years, and many individuals have encouraged the government to move ahead with a national NBS policy [26,29,30,31,32,33,34,35,36]. The issues impacting NBS implementation were previously detailed in a 2007 report on the Asia Pacific region [5], and many of these remain as issues still, as noted in a 2020 report on NBS issues in India: the impact of NBS of infant morbidity and mortality; the availability of related healthcare, particularly in rural areas; the lack of medical knowledge and education in the population; cultural influences; and cost [33]. In the listing of national healthcare priorities influencing government considerations, NBS continues to slowly increase in importance as the IMR decreases and approaches single digits [1,6], which increases the likelihood of government financial support. As an example, NBS for CH, CAH, SCD, and G6PDD are listed as examples of metabolic disease screening in India’s national child healthcare program enacted in 2013, Rashtriya Bal Swasthya Karyakram (RBSK) [37]. Recognizing the importance of state-specific child healthcare actions as part of RBSK, the Delhi government, through the Directorate of Family Welfare, adapted this health program in 2020. It was named Mission NEEV (Neonatal Early Evaluation Vision) and intended to provide universal NBS for CH and CAH and early diagnosis of visible birth defects, CCHD, NHS, and retinopathy of prematurity (ROP) [38].

Because of its size, India faces extreme challenges in implementing any public health program nationally. It is therefore prudent to approach implementation first at the state level, with expansion to other states and the country as successful implementation models emerge. Project models already exist in Chandigarh, Goa, and Kerala [33,36] and now in Delhi [26]. “The Chandigarh program, initiated in 2017, is concentrated in four urban hospitals. The Goa program, which is active in 13 government hospitals, was implemented in two phases to address early shortcomings and has increased focus on follow-up and treatment. The Kerala program, initially a pilot but now present in over 90 government hospitals, seeks to screen every birth in government hospitals with eventual expansion to private hospitals [33]. The Unique Methods of Management and Treatment of Inherited Disorders (UMMID) initiative by the Government of India, for NBS in selected aspirational districts, and the NEEV mission by the Delhi government are the newer public sector initiatives targeting genetic disorders and metabolic error screening.” [36]. The UMMID program has two components. The antenatal component addresses universal NBS for hemoglobinopathies and seeks to identify families at increased risk for a genetic disorder based on family history, including a long list of possible conditions such as Duchenne muscular dystrophy, Fragile X, spinal muscular atrophy, cystic fibrosis, lysosomal storage disorders, and others. Screening for these “other” conditions is not done routinely but, rather, based on suspicion and family history.

The major determinants for the effective implementation of universal NBS at the national level include (1) obtaining and evaluating case-finding data to determine the most appropriate screening panel, (2) developing and sustaining training for healthcare personnel, (3) educating the public and policymakers, (4) recognizing and harnessing the power of NBS for societal benefit, and (5) developing a national NBS policy [26].

### 3.4. Indonesia

Indonesia, officially the Republic of Indonesia, is the world’s largest archipelago, with over 17,500 islands covering a total area of 735,354 sq km with 34,000 miles of coastline (see Figure 2). With a total population of 281 million people in 2024, it is considered the fourth most populated country in the world and ranks fifth in births (4.62 million births annually). More than half of the population lives on Java, the world’s most populous island. Indonesia has an elected bicameral legislature and functions as a presidential republic. There are 38 provinces, with 9 having special autonomous governing status. Government subdivisions include provinces (with a legislature and governor); regencies (regents); and cities (mayor and legislature), districts, and villages (divided into neighborhoods and hamlets).

Following IAEA’s Regional workshop on NBS for CH in May 1999, and utilizing IAEA project support funding, two NBS projects for CH began in 2000 in Bandung and Jakarta [39]. The Bandung project was initiated in five maternity and two general hospitals. It aimed to estimate the local incidence of CH and to identify/evaluate obstacles to sustainable program implementation [40]. Initially, using cord blood was the specimen of choice, but high recall (3.3%) and inability to use cord blood for future expansion to other conditions resulted in changing to dried bloodspots in 2001 (extending to the remainder of the project; recall = 0.6%). Reported implementation challenges included obtaining parental permission for testing and tracking screen positive patients for confirmatory testing. A national NBS mandate was suggested, along with an improved patient recall system and better education for parents and healthcare professionals. Other than periodic project reports to IAEA, the results of the Jakarta project remain unpublished.

Following a health technology assessment in 2006, CH NBS was expanded to eight provinces in 2008. Thirty-eight more laboratories were added in 2014 in response to a decree from the Ministry of Health that all newborns should be screened for CH [41]. A 2015 study across five cities (30 hospitals): Denpasar (6), Banten (8), Jakarta (14), Semarang (1), and Yogyakarta (1) evaluated the feasibility and results of NBS for CH and CAH in Indonesia for possible guidance in future program implementation [42]. While the sample size was statistically inadequate to determine disease incidence, a relatively high occurrence of both conditions was observed (although further studies are needed for more precise predictions). Patient recall was noted to be a problem “due to difficulties in contacting parents and lack of awareness”. Nationwide NBS was considered feasible with three caveats: (1) “the MOH should recognize the importance of NBS through funding and integration of NBS in national health programs”, (2) there should be “campaigns to increase awareness amongst parents”, and (3) it is urgent that there be “good partnership with healthcare providers and stakeholders”. A 2023 comprehensive review of the current state of NBS in Indonesia and the challenges in its sustainable implementation nationally also noted some of these same issues. Further, it noted a lack of a number of items necessary to system development: “…prevalence data, ethical issues, infrastructure, cost–benefit analysis, logistical issues, government support, patient issues, a lack of commitments, and a lack of healthcare workers, specialization, and training.” [43].

Throughout the years, NBS coverage has remained low in Indonesia, reaching only 2.3% of newborns by 2022. In August 2022, CH NBS was relaunched, and the Ministry of Health issued a new decree (HK.01.07/MENKES/1511/2023) requiring CH NBS in order for healthcare providers to claim delivery reimbursement rates from the national health insurance [44]. This action led to a significant increase in CH NBS coverage. Eleven laboratories were capable of processing NBS in 2023, up from the original two laboratories when CH NBS was first initiated. In January 2023, 21,718 samples were received for CH NBS, and the number of specimens steadily increased throughout the year, with a notable surge from September onwards. By the end of the year, 1,249,094 specimens had been received (28% coverage).

From January to July 2024, the monthly number of samples consistently exceeded 150,000, after peaking at 257,575 in January. As of July 2024, 28.29% of the yearly target for specimens had been achieved. Also in 2024, pilot projects began screening for G6PDD, CAH, and CCHD. NHS is expected to begin in 2025, along with screening for vision loss and hyperbilirubinemia in 2026. As NBS coverage has expanded nationally, the MOH has added three additional regional laboratories, and the number is expected to total 29 by the end of 2025, including referral laboratories, regional health laboratories, and private laboratories.

Healthcare professionals in Indonesia continue working towards increasing awareness of NBS and developing a cost-effective, efficient approach to screening. A national cross-sectional electronic survey was conducted in 2023 focused on identifying and exploring the experiences of healthcare professionals and program administrators in CH NBS (from over a dozen professions). The identified issues included a lack of logistical and infrastructural support, lack of knowledge and training of healthcare professionals, and hesitation from families. Also noted were challenges related to refusal from families (39.2%), hospital discharge before 24 h (38.3%), and limited availability of filter paper (35.9%). Concerted efforts are needed “to improve the access to and availability of resources, increase the capacity for sample collection and analysis, educate and empower healthcare professionals, and develop educational materials and programs to promote the understanding and acceptance of NBS amongst families” [39].

### 3.5. Laos

Laos, officially the Lao People’s Democratic Republic, is landlocked in the heart of the Indochinese peninsula bordered on the northwest by Myanmar and China, on the east by Vietnam, on the southeast by Cambodia, and on the west and southwest by Thailand (see Figure 2). Slightly over half of the Laotian population are Lao people, who live mostly in the lowlands. Mon-Khmer, Hmong, and indigenous hill tribes inhabit the foothills and thickly forested mountains. Laos has one of the fastest growing economies in the region. It is divided into 17 provinces and one prefecture that includes the capital city, Vientiane, with the provinces further divided into districts and then villages (urban villages are essentially towns). The population is approximately 7.6 million, with ~160,000 births annually. The median age of the population, 21.6 years, makes it one of the youngest populations in Asia. Professional resources are scarce, with only 206 pediatricians and 4 neonatologist serving the population.

The first NBS efforts in Laos arose following neonatal teaching sessions by a German University held at the University of Laos in Vientiane between 2004 and 2006 [funded by a German non-government organization (NGO)]. Lack of knowledge about NBS by course attendees led to planning a pilot study to determine the frequency of CH and CAH in Vientiane. The project was designed to establish a bloodspot collection system and to demonstrate the feasibility of tracking and follow-up for NBS screen positive results. Laboratory methods for a variable number of screening parameters were planned, with expansion to other geographic areas expected over time. Diseases were selected for screening that could be successfully managed locally and were affordable to the majority of the population [45]. The resulting pilot occurred in 2009–2010 and initially included specimens from four Vientiane maternity hospitals [Mahosot Hospital (119), Sethathirath Hospital (1653), Friendship Hospital (1134), and Mother and Child Health Hospital (6866)]. Four other Laotian hospitals joined over the course of the project [Vientiane Provincial Hospital Maria Theresa (893), Champasack Provincial Hospital (447), Borikhamxay Provincial Hospital (131), and Khammouane Provincial Hospital (119)].

Specimens (*n* = 11,362) were collected prior to hospital discharge (86% < 24 h after birth) and air-shipped weekly to the Hamburg, Germany screening laboratory. Routine early hospital discharge led to higher recall in Laos compared to Germany. Difficulty with local physicians not following retesting protocols was identified as a challenge. Recommended recall testing was often avoided when the screen value was just outside of the reference range. Nonetheless, the feasibility of NBS in Laos was demonstrated with CH and CAH frequencies appearing similar to the rest of the world, despite the low numbers of specimens analyzed. Confirmed cases included two infants with CH and one with non-salt wasting CAH [45,46]. One potential case of salt wasting CAH with loss of function mutations in both alleles showed no clinical affects over a 6-year period. This unusual case led its investigators to speculate that “…an unknown percentage of infants with so-called obvious classic CAH may have sufficient intrinsic 21-hydroxylase activity; hence, they do not suffer from SW crisis and/or early neonatal death.” [46].

A second pilot was initiated in January 2019 through July 2020, again with technical support from Germany but with laboratory services provided locally in the laboratory of Mother and Newborn Hospital in Vientiane [47,48]. This pilot aimed to continue studies of CH incidence; refine blood specimen collection within Vientiane; and improve the processes for recall, confirmatory testing, and patient management of screen positive newborns. Educational workshops were offered to obstetricians, pediatricians, nurses, and midwives from the six participating hospitals. Topics included (1) history of newborn screening; (2) organization of sampling, transport, and testing; (3) documentation and communication of results; (4) follow-up of positive tests; and (5) counseling parents. Particular emphasis was placed on the importance of accurate and complete patient data.

This project was intended to mark the beginning of a permanent NBS program in Laos. Start-up funding provided by the German NGO was based on a commitment from the Ministry of Health to take over funding at the end of the German funding period. Treatment costs with L-thyroxine were affordably low (0.01 USD/day). Challenges included a lack of awareness of the importance of NBS by physicians and nurses, the mechanism for obtaining parental consent, and the transporting of specimens between hospitals. Consideration was given to awareness campaigns, possible financial incentives, education on the consent process, and hiring a driver for specimen transport. Future plans included NBS expansion to provincial and district hospitals, with knowledgeable birth attendants expected to assist in specimen collection at health stations in remote areas of the country [48]. Unfortunately, NBS was discontinued in January 2024 when German funding ended and collection/analysis supplies were depleted. Considerations/discussions are ongoing in the Ministry of Health and the Pediatric Association, with possible funding assistance from UNICEF. Formation of a national NBS committee is a possibility.

### 3.6. Mongolia

Located between Russia and China, Mongolia is a landlocked country of 1.56 million sq km, with 67.1% of the 3.5 million people living in the capital city of Ulaanbaatar (see Figure 2). There are approximately 67,000 births annually, and the growth rate is ~2%. Iodine deficiency in Mongolia is mild to moderate, with considerable goiter present. A ten-year study of CH between 1989 and 1999 showed a prevalence of 1:3057 [49].

With the goal of developing a national NBS program for CH, a pilot NBS was initiated in 2000 by the Department of Nuclear Medicine, National University of Mongolia, Ulaanbaatar, Mongolia, with specimens collected at two of the maternity houses in Ulaanbaatar. Long-term goals included (1) implementation of a NBS program for CH in Ulaanbaatar; (2) creation of a national NBS mandate; (3) coverage of the full newborn population; (4) creation of a program for educating policymakers, health personnel, and the general public; (5) identification of sources to assist with finances; (6) initiation of a bulk reagent contract for NBS; and (7) participation in international quality assurance activities. With limited financial and training support from the IAEA, 1529 newborns were screened for CH from July 2000 to September 2001 using DBSs collected at 3–4 days of age. Immunoradiometric laboratory procedures were used in keeping with IAEA guidelines. Two newborns with elevated TSH/low T4 were diagnosed and treated for CH [49].

Implementation of the national NBS program for CH began in 2012, and the CH prevalence observed between 2012 and 2020 was 1/2091, but further studies were considered necessary to accurately assess the CH prevalence [50]. Currently, CH NBS in Mongolia is occurring in nine districts of Ulaanbaatar (eight maternity hospitals) and five Aimags (rural administrative subdivisions—twenty-one exist) and, between 2012 and 2024, has included the screening of over 40,000 newborns (~4.7% of the newborn population). Program infrastructure and operational guidance is based on the material published by the IAEA for use by its CH NBS projects in countries with developing economies [8]. Since 2012, limited assistance has been provided by Revvity [(formerly Perkin Elmer); Turku, Finland] in the form of equipment, technical support, and diagnostic reagents. For screening, dried blood spot (DBS) specimens were collected 24 to 72 h after birth and analyzed by the Revvity DELFIA TSH assay. Table 2 provides summary NBS data since 2000. During different time periods, funding sources have varied: IAEA (2000–2002), Mongolian Science and Technology Foundation (2012–2014), Rotary Foundation (2016–2023), and UNICEF (2023–2024).

Expanded screening for CAH began in 2012, and twenty-three cases of CAH were confirmed between 2012 and 2023 while screening 39,198 newborns (Table 2). This high prevalence (1:1452) may indicate a genetic predisposition similar to that previously reported for Yupik Eskimos in Alaska [51]. A master’s degree study of the clinical forms and early manifestations of CAH on 2957 newborns in the neonatal intensive care unit found one newborn with simple virilizing CAH [52]. CAH studies continue.

A 2015 report of a pilot study for G6PDD (Victor 2D; Revvity, Turku, Finland) in 205 newborns showed positive screening results in 63 (23.5%): 33 males (52.4%) and 30 females (47.6%), with jaundice lasting up to 2 weeks [53]. Based on these data, screening for G6PDD began in 2023 (Table 3), and the number of screen positives has varied from 3.4% in 2023 to 4.1% in 2024 or 3.7% overall. Currently, a second screen on recalled patients serves for confirmation while screening and diagnostic protocols are being refined. The current calculated prevalence for G6PDD is 1:380. As indicated, no cases of CF or GALT have yet been confirmed.

To maintain stability in the NBS system as health ministers change, the Minister of Health of Mongolia approved the “Guidelines for Newborn Screening on Dried Blood Spots” by Ministerial Order No. A/159 on 22 March 2022. Included in this order was formation of The National Integrated Newborn Screening Team to manage six types of screening: DBS, hip dislocation ultrasound, neonatal cranial ultrasound, retinopathy of prematurity, CCHD, and NHS. Inclusion of NBS in the national health insurance is currently under consideration, with anticipation of its approval soon, which will make NBS available to the entire newborn population [54].

During the past few years, considerable attention has been paid to advocacy and training. Online and in person training has been provided to over 100,000 persons, printed materials have been developed and distributed, and social media advertisements have been widely shared. The undergraduate and postgraduate training programs at MNUMS now include NBS in their curriculum. Core NBS group members received long- and short-term training in Finland, Russia, Kazakhstan, and Uzbekistan. Trainers from the Mongolian Society of Newborn Screening continuously train medical professionals throughout Mongolia, providing them with an Official Certificate of the Health Development Center upon completion of training. Guidance materials have been developed for training both health professionals and the public. NBS staff members have actively participated in international and domestic conferences and have published more than 50 articles and abstracts, including doctoral and master’s theses. Five branch centers for NBS have now been established in five regional diagnostic and treatment centers regionally distributed across the country.

### 3.7. Myanmar

Myanmar, officially the Republic of the Union of Myanmar (previously Burma), shares its borders with China, Thailand, India, Bangladesh, and Laos and has 1760 miles of coastline, bounded on the west by the Bay of Bengal and on the south by the Andaman Sea (see Figure 2). The country is divided administratively into the Nay Pyi Taw Council Territory and 14 states and regions. It consists of 74 districts, 330 townships, 398 towns, 3065 wards, 13,619 village tracts, and 64,134 villages. Myanmar has a steadily increasing population of approximately 51 million, with 30% living in urban areas, and there were 640,000 births in 2023, down from a high of 885,000 in 2019, at least partially due to the COVID-19 pandemic.

The country is currently recovering from years of political unrest, and the degree of governmental interest in NBS is unknown. While the original East Asia IAEA projects included Myanmar, participation in workshops over the years has been limited, resuming this year in Kathmandu. There appears to be considerable interest in NBS among health professionals and better educated parents who are aware of NBS, with particular interest in CH, G6PDD, NHS, and CCHD. While the incidence of CH in Myanmar is not known, it is likely to be high based on studies in the mountainous regions of Northern Thailand. In 2012, clinical practice guidelines for the recognition, diagnosis, and treatment of thyroid disorders were developed by a multidisciplinary panel of experts. These guidelines were intended to standardize the management and delivery of equitable and patient-centered care for people with thyroid function disorders. Part of these practice guidelines stated that “All newborn babies should be screened for congenital hypothyroidism by measurement of bloodspot TSH using a sample collected within 2–7 days after birth, as part of a national screening programme. The measurement of thyroid related hormones as part of a neonatal screening programme should be restricted to specialist laboratories and should have a turnaround time of <5 days. … All hypothyroid neonates should be treated … within the first 18 days of life.” [55]. The net result of these guidelines appears to be limited NBS for CH in some private hospitals.

Interestingly, The National Strategic Plan for Newborn and Child Health Development (2015–2018) [56] noted that “There will be community-based newborn screening through health volunteers at home visits and facility based newborn care by existing health manpower.” Information on the implementation of this plan is not currently available (see ref. [57], p.50, Section 7.5.1 Thrust Area 1: Newborn Care).

The presence of G6PD deficiency in the neonatal population has been documented in several studies, and neonatal jaundice was found to be the leading cause of morbidity (23.8%) and one of the top 10 causes of neonatal mortality (1.6%) reported in hospitals in 2018 [57]. Screening for G6PD is currently ongoing in all maternity and tertiary care hospitals, and in some private hospitals, for both male and female newborns with early or significant jaundice. The results of a study of the contribution of genetic factors to high rates of neonatal hyperbilirubinemia on the Thailand–Myanmar border in 2022 illustrated that genetic risk factors in this population play a large role in neonatal jaundice. Further, it noted that improved diagnostics and different screening strategies are urgently needed [58]. To this end, a number of studies have assessed various methods for screening newborns with the goal of finding the most efficacious methodology [59,60,61].

Some NBS exists in the private sector for G6PD, CH, NHS, and CCHD. There is currently no NBS pilot or program. Efforts are ongoing to identify and assemble a leadership team to develop a program, first at the local level and later expanding to national coverage. G6PDD screening is currently focused on all newborns with early or significant jaundice in maternity and tertiary care hospitals and in some private hospitals.

### 3.8. Nepal

Nepal, officially the Federal Democratic Republic of Nepal, is a landlocked country bordered to the north by China and to the south, east, and west by India (see Figure 2). Its diversified landscape varies from the lowland terai plains in the south to hills in the center and the towering Himalayas, home to Mount Everest, in the north. Nepal is a federal democratic republic, with Kathmandu as the largest city and capital. It is known for its beautiful landscapes, vibrant culture, and rich cultural legacy, which includes numerous UNESCO world heritage sites. The population of Nepal is around 31 million, with approximately 600,000 annual births. The country is culturally diverse, with more than 140 ethnic groups and castes and over 120 languages. Urbanization is increasing as people seek better living conditions and employment; however, more than half of the population currently work in agriculture. A salt iodination effort across the country was implemented some time ago to combat iodine deficiency in the mountainous regions and is assumed to have been successful in reducing the prevalence of CH.

The prevalence of conditions typically included in NBS programs is largely unknown in Nepal. However, the presence of hemoglobinopathies and G6PDD on the Indian subcontinent have been reviewed and provide possible conditions for screening consideration [62]. A 2010 review of IMDs in Nepal noted that studies of the prevalence and treatment of IMDs in the Nepalese population were lacking, resulting in conflicting statistics and the need for more pilot studies. A national screening program was suggested as a next step based on pilot testing results [63]. A 2019 report reviewed over 440 previously published metabolic case reports and concluded that, in addition to CH and hemoglobinopathies (HGB), including SCD, the prevalence of G6PDD, Wilson’s Disease, and lysosomal disorders was sufficient for consideration for NBS [64].

Two recent reports, one in 2019 [65] and one in 2024 [66], reviewed pilot projects aimed at assessing the need for, and feasibility of, NBS in Nepal. A review of the population demographics and ethnic background of the Nepalese people can be found in the 2019 report and is not repeated here. That pilot, a collaboration between Kathmandu Medical College and Teaching Hospital and the Swiss Newborn Screening Laboratory in Zurich, sought to identify IMDs that might be prevalent in Nepalese children, thereby benefiting from NBS. A total of 4360 bloodspot specimens from a newborn population comprised of 78% Aryans, 11% Newars, 10% Mongols, and 0.5% Tharus, the four most prominent ethnic groups, were collected and sent periodically for analysis in Zurich using the Swiss NBS panel of screening conditions and analytes [thyroid-stimulating hormone (TSH), total galactose (tGAL), galactose-1-phosphate uridyl transferase (GALT), 17-α-hydroxyprogesterone (17-OHP), immunoreactive trypsinogen (IRT), phenylalanine (PHE), tyrosine (TYR), leucine (LEU), isoleucine (iLEU), valine (VAL), glutarylcarnitine (C5DC), and octanoylcarnitine (C8)]. While the sample size was small and the *terai* plains and high mountain populations were underrepresented, two cases of hypothyroidism and one case of cystic fibrosis were identified. These cases suggested that CH and CF should be considered for NBS. A well-documented high incidence case finding data for sickle cell disease in the Tharu population also led to the suggestion to that HGB NBS might also be appropriate (even though HGB testing was not part of the Swiss screening panel used for the pilot study) [65].

The 2024 report described a pilot between November 2021 and May 2022, which sought to determine the feasibility of NBS and its acceptability among healthcare providers and parents [66]. This pilot included 825 consenting parents (of 835 approached) at Paropakar Maternity and Women’s Hospital (PMWH), Kathmandu, the largest maternity hospital in Nepal (~24,000 births annually). Heelstick specimens were collected between 24 and 48 h after birth and sent three to four times/week to a private screening laboratory in India for testing of six candidate conditions, which could be confirmed and managed locally (CH, CAH, G6PDD, galactosemia, SCD, and biotinidase deficiency (BIO). Certain high-risk patients were also screened for additional grouping conditions that included disorders of fatty acid oxidation, organic acid, amino acid, lysosomal storage, severe combined immunodeficiency, and carnitine deficiency). Staff were trained on dried blood spot collection, transport protocols and performance metrics were established, and surveys were developed to determine acceptability among healthcare providers and parents.

Based on the successful operation of the NBS project and the detection of screened conditions in 13 newborns [CH = 3, G6PDD = 2, CAH = 1, elevated acylcarnitine = 1, BIO = 1, and 5 HGB carriers (4 Hb A,E; 1 Hb A,D)], the study authors considered NBS to be feasible. Of 116 healthcare providers surveyed, 67% reported a moderate understanding of NBS, and all indicated that screening was beneficial, with early diagnosis and treatment noted as significant benefits. Identified challenges included cost, unavailability of screening laboratories, and difficulties in contacting families for follow-up of positive screening results. Parents overwhelmingly considered a “no cost” NBS option as the most important factor in choosing to screen. A government-sponsored program was considered to be a key factor in establishing sustainable NBS in Nepal [66].

In August 2024, a small budget was allocated by the MOH to begin NBS at PMWH, and Phase I implementation has begun. Screening laboratory equipment was donated, along with supplies sufficient for 2500 tests. A NBS Committee of nine persons has been formed at PMWH to oversee infrastructure development and program implementation. Personnel from the Department of Pathology, PMWH, have received training from the equipment manufacturer, and nursing staff and medical officers have received orientation regarding specimen collection. Successful implementation efforts are expected to result in NBS expansion to other hospitals and provinces as infrastructure stability is demonstrated. A meeting of the workshop coordinators and the Minister of Health during this conference confirmed the support of the Minister of Health for newborn screening for all Nepali newborns.

### 3.9. Pakistan

Pakistan, officially the Islamic Republic of Pakistan, is the fifth most populous country in the world, with a population of approximately 241 million, with the second largest Muslim population (Indonesia is larger). There are approximately 6.4 million births annually in Pakistan (fourth globally), and its population median age of ~19 makes it one of the youngest populations in Asia. It shares land borders with India, Afghanistan, Iran, and China; a maritime border with Oman in the Gulf of Oman; and is separated from Tajikistan in the northwest by the narrow Wakhan Corridor (see Figure 2). Pakistan is a federal parliamentary republic that consists of four provinces (Punjab, Khyber Pakhtunkhwa, Sindh, and Balochistan) and three territories to the north and northeast (Islamabad Capital Territory, Gilgit-Baltistan, and Azad Kashmir). It has two megacities, Karachi and Lahore, and over 65% of the population resides in urban environments.

The first reported pilot study of NBS for CH in Pakistan was conducted from March 1987 to September 1988 [67]. Five thousand newborns delivered at Aga Khan University Hospital (AKUH) and four maternity homes in metropolitan Karachi were included in the study. The CH prevalence observed was 1:1000, which is higher than the global average, but the reason was not determined (iodine deficiency is a problem in the mountainous regions of Pakistan but not in the coastal region around Karachi). A 20-year review of CH cases at AKUH found that, overall, the incidence of CH appeared to be about 1:1600, but there were gaps in the data reviewed [68]. A cross-sectional descriptive study was started at Shaikh Zayed Hospital, Lahore, and expanded to Jinnah Hospital, Lahore, and after eight months, two cases of CH were found in 1357 screened newborns [69]. While the sample size was too small to determine true prevalence, the apparent CH frequency in these studies points to a need for NBS.

The current NBS efforts in Pakistan are fragmented, with various efforts ongoing to initiate provincial screening programs. With support funding from the Asia Pacific Regional NBS Project of IAEA, the Pakistan Atomic Energy Commission (PAEC) took steps to participate in IAEA training activities aimed at implementing a NBS for CH beginning in 1999. A small project was initiated that included the development of parent education materials and informational posters encouraging parents to consent to NBS. The IAEA hosted regional training for personnel from Pakistan and other Asia Pacific projects from 1999 through 2005 and supported travel expenses for expert onsite consultation. Despite these efforts, a sustainable NBS program was never institutionalized. After IAEA funding ceased in 2008, a country representative continued to participate in volunteer NBS workgroup activities and work towards implementing a sustainable national NBS program in Pakistan.

In developing countries, NBS services sometimes develop independently and simultaneously in different locations within a country. This appears to have occurred, and may still be occurring, in Pakistan. While NBS for CH was initiated as a project of PAEC in 1999, published reports about NBS in Pakistan make no mention of this project. As an example, a report of a March 2019 Multidisciplinary Conference on Newborn Screening for Rare Disorders in Pakistan noted the existence of NBS only at a Karachi-based private hospital that offers NBS for five conditions (CH, CF, CAH, BIO, and GAL) and through a local NGO [70]. At this conference, led by the Pakistan Society of Chemical Pathology and hosted by Aga Khan University (AKU), a working group, the Pakistan Inherited Metabolic Disease Network (Pak-IMD-Net), was organized to improve the diagnosis and management of IMDs in Pakistan, including advocating for inclusion of IMDs in a national NBS program. A newsletter from the working group in 2022 [71] noted that educational workshops for healthcare professionals have been held that stress the clinical utility of NBS, the tests available, and interpretation of screening test results. Issues related to screening laboratory protocols (i.e., cutoffs) were noted, along with development of a virtual learning system for training in bloodspot collection techniques. A NBS program was reportedly launched in 2021 at a central hospital facility in Hyderabad and expanded to secondary hospitals in 2022. NBS using advanced laboratory techniques was launched at the Karachi AKU Hospital in 2023, allowing detection of a wider range of rare diseases.

On an unrelated parallel tract, a NBS law and prefectural screening program were developed in Sindh Prefecture. The Newborn Screening Act of 2013 [72] was officially implemented on 1 January 2020. Its main objective was/is to ensure that all newborns in Sindh are screened for certain disorders that can be treated or managed with early intervention. The Act established a legal framework for establishing NBS in Sindh, including developing and implementing guidelines and protocols for screening. NBS was required at 24–72 h after birth unless refused in writing by the parents (excluding babies in intensive care, who must be screened by 7 days of age). It also established an advisory committee on newborn screening. Its apparent success was applauded in a news release in 2021 [73], which noted that 75,000 newborns were screened within the first 18 months after its commencement on 1 January 2020. Of 2200 screen positives, only 700 (about 32%) were able to be followed (170 had died, and the rest did not respond). Of these, forty-five were diagnosed with CH. The poor follow-up rate was attributed to a lack of knowledge about the importance of screening by parents, who were unwilling to have their screen positive newborn retested, since there were no symptoms.

A series of reports published since 2018 as part of an ongoing research project, “Diagnosis of treatable inborn metabolic disorders of intellectual disability”, discuss the history of various inborn metabolic disorders (IMDs), increased case findings due to consanguinity, related health issues, and the possibility of early detection and treatment through NBS in Pakistan [74,75,76,77,78,79]. The prevalence of IMDs in Pakistan is generally not known, but their detection through population-wide NBS programs in other countries is well documented. Reference to the benefits that could be realized in Pakistan by implementing a national NBS program is a common theme throughout these reports. Policymakers were urged to establish NBS in Pakistan based on three critical benefits: (1) a healthy lifetime as a result of timely diagnosis and early treatment, (2) reduced family suffering, and (3) minimal burden to society and the healthcare system [79].

In 2020, a review report described the relatively high frequency of CH observed clinically in Pakistan. It noted the pressing need to carry out systematic prospective studies to determine the true incidence of CH and other screenable conditions in Pakistan. Prevalence studies are needed to help health policymakers understand and determine the need for universal or expanded NBS across the country [80]. A 2022 literature review of the CH prevalence studies in Pakistan found nine related publications, which together led to a projected incidence of 1:1600, higher than the global average of approximately 1:2500, possibly due to iodine deficiency in parts of the population. This high CH incidence supports the need for NBS for CH, which leads to improved outcomes for early detected and diagnosed newborns [81]. Similarly, another 2022 report reviewed the available data on CH and five other conditions and concluded that the six conditions, CH, CAH, BIO, GAL, G6PDD, and SCD, should be part of a NBS panel in Pakistan [82].

Despite the lack of a national NBS in Pakistan, a number of researchers continue with efforts to provide NBS-related laboratory data helpful in establishing national norms, including (1) a cross-sectional study at the Armed Forces Institute of Pathology, Rawalpindi, Pakistan, on NBS DBSs collected from healthy newborns at 2–6 days from May to November 2019, to determine the reference interval of BIO activity in healthy neonates [83]; (2) a multi-center cross-sectional validation study at Zahra Beau Naqvi Foundation Welfare Trust laboratory, Islamabad, Pakistan, from September 2016 to September 2018 using heelprick specimens from neonatal units of 39 hospitals based in Punjab, Khyber Pakhtunkhwa, Gilgit-Baltistan, and Azad Jammu and Kashmir to validate TSH screening using DELFIA technology [Dissociation-Enhanced Lanthanide Fluorescent Immunoassay; Revvity (formerly Perkin Elmer), Turku, Finland] [84]; (3) a study in collaboration with the University of Iowa (USA) to determine reference intervals and to evaluate the effect of gestational age, gender, weight, and time of sampling on amino acids, acylcarnitines, and succinylacetone in NBS DBS specimens between November 2017 and February 2019 [85]; and (4) an audit study of short-term results reporting for screen positive results from January 2015 through December 2016, showing an improvement from 62% to 77% for results conveyed to the general pediatrician or neonatologist after identification and corrective measures were taken to address the lack of policy awareness, lack of computer emphasis (highlighting) of screen positive results, and absence of routine result monitoring [86].

At least two studies involving parents have been recently reported: (1) a study from January 2012 to August 2013 at Aga Khan Hospital for Women at Garden East in Karachi, Pakistan, which showed that health education to improve mothers’ knowledge and attitudes towards having their newborns screened for CH resulted in an increased acceptance of NBS (78.9% vs. 57.7%) [87]; and a study of newborns screened for CH at Shaikh Zayed Hospital, Lahore, Pakistan, from March 2018 to April 2019, demonstrating a NBS acceptance of 57.3% vs. 41.1%, with proper counseling of both parents prior to obtaining NBS consent vs. awareness through printed brochures [88].

Studies published in 2024 include a retrospective chart review of IMD patients in the Indus Hospital and Health Network (IHHN), Karachi, from October 2020 through March 2022, in order to determine the extent of screening and the need for extended workups related to local NBS using tandem mass spectrometry [89] and a review of organic acid disorders, their presence in Pakistan, and the value of NBS in closing the health equity gap in developing countries [90]. This report summarizes the current status of NBS in Pakistan as follows: “Unfortunately, developing countries, including Pakistan, lack basic health facilities and have no local or national NBS for any of the inherited metabolic disorders. The implementation of effective programmes is crucial for reducing morbidity and mortality, and for improving the quality of life for the affected children and of their families, thus promoting global health equity”.

### 3.10. Vietnam

Vietnam, officially the Socialist Republic of Vietnam, has a long land border of 4550 km, bordering China to the north, Laos and Cambodia to the west, and the Eastern Sea (South China Sea) of the Pacific Ocean to the east (see Figure 2). It has a diverse topography of 75% hills and low mountains and 25% deltas, with a resultant humid climate and coastline of 3260 km. It has a long land border of 4550 km, which borders China to the north; Laos and Cambodia to the west; and shares maritime borders with Thailand (Gulf of Thailand) and the Philippines, Indonesia, and Malaysia (South China Sea). The population of Vietnam is approximately 100 million, making it the 15th most populous country. The number of births in 2023 was ~1.2 million.

The first formal NBS activities in Vietnam began with its IAEA project beginning in 1998 at the National Children’s Institute. It became national in scope in 2006. Since 2015, NBS has been available to every child born in a public healthcare facility. The program includes three diseases (G6PDD, CH, and CAH) with specimens collected 1 to 7 days after birth. In 2018, the Department of Population and Family Planning of the Vietnam Ministry of Health reported that the percentage of newborns undergoing NBS in 2018 was 38.5% of births [91]. This report also notes the lack of structured economic evaluations of NBS in LMICs to determine the best way forward and uses Vietnam NBS as the case study.

A multi-national study report in 2018 examined the diversity of organic acidemias, fatty acid oxidation disorders, and amino acid disorders in Asian countries. Selective screening for IMDs was performed using gas chromatography-mass spectrometry and MS/MS. Between 2000 and 2015, 39,270 patients in Asian countries (Japan, Vietnam, China, and India) were tested for IMDs. Β-Ketothiolase deficiency (BKT) was found to be particularly frequent in Vietnamese (33 out of 250 IMD cases) [92]. A report on the clinical phenotypes, biochemical and molecular characteristics, and outcomes of 41 BKT patients detected between 2005 and 2016 at National Children’s Hospital (Hanoi) has also been published. While the incidence of BKT worldwide is estimated to be ~1:1,000,000, the calculated incidence in Vietnam is ~1:190,000, which supports its inclusion in NBS [93]. A study of five patients with mucopolysaccharidosis type I (MPS-I) in the Kinh ethnic population, all from unrelated families in the same small community with the same previously unreported variant, indicate a probable founder effect and increased population incidence. These data support the inclusion of MPS-I in Vietnamese NBS [94].

In December 2020, the Prime Minister approved expansion of the prenatal and NBS programs until 2030.These programs exist in 10,000 districts and communes with NBS performed using heel prick specimens. NBS coverage in 2019 was reported to be 40%. The goal of the NBS expansion is to test 90% of newborns for at least five of the most common congenital conditions in the next ten years. Under the expansion program, facilities to provide NBS will be established in 90% of the communal-level localities nationwide. NBS and diagnostic facilities are also expected to be developed at hospitals in 56 provinces and cities. Five regional screening and diagnosis centers will be upgraded by 2025, and two new centers will be built in the northern midland and mountainous region and in the Central Highlands. To date, low awareness about the benefits of NBS, especially in ethnic minority areas, have hindered the efficiency of the program, but efforts to improve the situation are underway [95].

A recent study in the Obstetrics Department at the Central Maternity Hospital sought to determine the current state of knowledge among mothers regarding heel stick blood specimens and to analyze the decision-making process for NBS. In cases where prenatal counseling had occurred, 72.4% of mothers agreed to have their newborn screened. However, despite 100% of mothers receiving counseling after delivery, only 40.5% correctly understood the purpose of the heel stick. The authors note that post-natal counseling is likely not a good idea due to the stresses and confusion of the birthing process, and they conclude that “It is crucial to strengthen counseling efforts on newborn screening both before and after childbirth, and further research is needed to understand why some mothers are hesitant to have their child undergo newborn screening.” [96].

Table 4 summarizes data demonstrating the impact of NBS since 2021 in Vietnam as the program has been institutionalized. Information on the rate of compliance in receiving confirmatory testing is not yet reliably available, and thus, the indicated prevalences are likely low, particularly for G6PDD, which has the highest number of screen positive results. Efforts are currently underway to improve the reporting process, including defining the elements most useful in defining the efficacy of the NBS process.

## 4. Discussion

This APSHG Working Group of NBS leaders in Asian countries with less than 50% NBS coverage continues to strive for sustainable national NBS in each country, despite significant political and economic challenges in some. Initial messages of hope and encouragement for successful NBS implementation/expansion were delivered by regional and international health leaders: Anshu Banerjee, Director, Maternal, Child, Newborn and Adolescent Health and Ageing; Honorable Budi Gunadi Sadikin, MOH, Republic of Indonesia; Honorable Teodoro Herbosa, Secretary, Department of Health, the Philippines; Brian Chung, President, APSHG; and Aman Pulungan, Executive Director, International Pediatric Association (IPA). Attendees were specifically challenged by the WHO to continue towards sustainable NBS in step with the 21 June 2024 resolution at the 77th World Health Assembly of WHO, entitled “Accelerate progress towards reducing maternal, newborn and child mortality in order to achieve Sustainable Development Goal targets 3.1 and 3.2” [97]. Messages from the heads of the health ministries in both Indonesia and the Philippines provided examples of the commitments needed to formally institutionalize nationwide NBS and achieve at least 50% coverage by 2025 (Indonesia) and to expand NBS to include 29 screening conditions with over 95% newborn coverage (the Philippines). Messages of support from the APSHG and the IPA emphasized the value of professional organizations in supporting national NBS initiatives.

With these introductory messages as a backdrop, conference attendees embarked on two full days of reviewing the current state of their NBS efforts, presenting data illustrating their accomplishments, reviewing challenges encountered in implementing sustained NBS, and setting goals for the next three years. Since membership in the working group is predicated on the availability of NBS to fewer than 50% of newborns in the jurisdiction represented, members “graduate” when this goal is met. Thus, as noted in the Introduction, while the group originally included members from China, Korea, Malaysia, the Philippines, Thailand, and Sri Lanka, NBS in each of these countries now reaches >90% of the newborn population, and these countries have “graduated”. Graduates continue to be available to consult on strategies to improve and expand NBS in other countries. At the current conference, representatives from the Philippines NBS program, the last to graduate, were invited to participate with updates on their successes and input on the topic of quality assurance.

**Figure 4:**
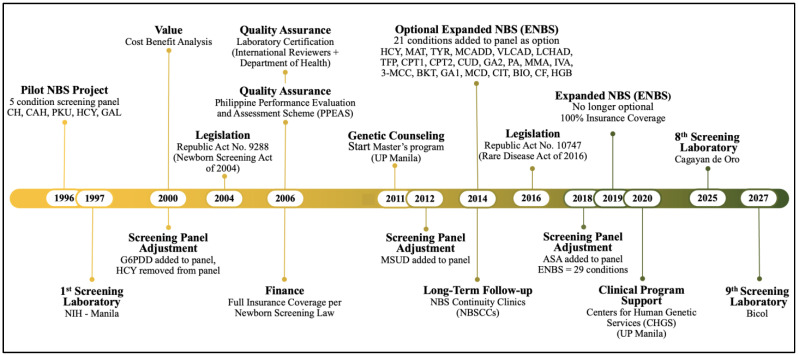
Timeline for implementation and expansion of the Philippines newborn screening system—1996–2027.

The Philippines NBS program has grown from a beginning NBS research project, screening and collecting incidence data for five conditions in 24 hospitals in Metro Manila in 1996, to a nationwide mandate for 29 conditions covering >95% of Filipino newborns in over 7100 birthing facilities [98]. While the initial project operations budget required a small payment from parents, the current expanded program is fully covered by government insurance. A timeline is presented in Figure 4 that can serve as a model for developing programs.

From a beginning that included screening laboratory services from the New South Wales Australia NBS program for the first year, the Philippines NBS program now enjoys the services of seven screening laboratories and the follow-up services of 33 continuity clinics, all of which are strategically placed throughout the country. The net result is the detection of almost 300,000 cases of conditions of varying clinical significance since the program began. Table 5 provides a partial listing of conditions detected through the years. Variations in the number screened across conditions reflects different screening start dates, as indicated in the table footnotes. NBS for hemoglobinopathies and thalassemias began in 2014, as did NBS for inborn errors of metabolism using tandem mass spectrometry [99,100]. During the 2014–2023 time period reported for hemoglobinopathies, 65,821 Hb E-carriers (HbF,A,E) and 1678 Hb S-carriers (HbF,A,S) were reported. Articles recently published have described tyrosinemia-1 and cystic fibrosis case findings [101,102]. Two additional cases of cystic fibrosis have now been confirmed using molecular analyses, along with four cases of sickle cell anemia [homozygous form of sickle cell disease (SCD-SS)].

Aside from discussions on current NBS operations, challenges, and future plans, workshop attendees focused their attention for a half-day discussion on the NBS system and the importance of ensuring quality services system-wide. In particular, consideration was given to the 6-part NBS system: education (parent, professional, and policymaker); screening; short-term follow-up/tracking; diagnosis; management/treatment; and long-term follow-up/quality assurance [103]. Focus was given to the comprehensive Performance Evaluation and Assessment Scheme (PEAS) developed in the USA; its component parts (pre-analytical, analytical, and post-analytical); and how it can be modified for use in developing NBS systems [10]. PEAS adaptations and its utilization in the Philippines and elsewhere were reviewed with the goal of providing ready resources for use in new and expanding programs. While the original PEAS provides definitions and operational details of a comprehensive listing of NBS system components (i.e., the “gold” standard), developing programs are left to pick and choose the elements applicable in their own environment. The manner in which the Philippines NBS program has used the USA model to develop and utilize Philippines PEAS provided attendees with a usable, real-life example for their consideration [11,12].

Having presented the current status and challenges in their own program, attendees listened to presentations from other programs, engaged in discussions of the issues facing each program, and evaluated and discussed how best to develop system-wide quality assurance, and workgroup members were paired (new programs mentored by more established programs and/or international consultants) for goal-setting discussions. Each participating country created three-year work plans with achievable goals for reassessment at future workgroup meetings. A short presentation to the full group by individual country representatives allowed the workgroup to understand the near-term goals and expectations for NBS in each country. The open forum allowed all participants to comment on the various work plans, and presenters used the discussions to refine their proposed plans.

Workshop participants agreed that the primary challenges impacting NBS effectiveness are similar in most developing programs: (1) resource constraints/management (funding and logistics regarding specimen collection and testing supplies); (2) screening laboratory infrastructure (technical capability, capacity, and location); (3) follow-up and treatment (efficient, effective, and timely tracking and case management); (4) geographic and demographic diversity (remote and rural areas have limited access to healthcare facilities and infrastructure); (5) education and awareness (affecting refusal for testing); (6) early hospital discharge of newborns (requiring procedures and protocols to assure screening); (7) poor specimen quality (requiring continuing training for specimen collectors); (8) data management/coordination (robust system for data collection, analysis, sharing, and storage); and (9) cultural and ethical considerations (attention/balance of parental and child rights). We previously noted some of these same issues in our earlier report on consolidation efforts in the region and noted some of the steps that could be taken to overcome these challenges [9].

While NBS programs can develop in settings outside of the public health system, for example, in academic settings, the ultimate goal of sustainable and universal access can only be attained within, or in collaboration with, the public health system. In general, the work plans provided local details for overcoming the challenges perceived as slowing progress towards national NBS implementation and sustainability. For example, participants identified various local meetings at which to present NBS information; identified influencers to be educated and enrolled for assistance in educating professionals, policymakers, and/or the public; and set timelines for completion of self-assigned tasks. Experiences with national health insurance systems and stepwise approaches to the inclusion of NBS in insurance benefits were also the subject of considerable discussion and planning efforts.

A summary declaration was prepared by meeting participants to formally recognize and document the workshop and its collaborative activities. Similar to declarations from the first two workshops in Cebu and Manila previously published [9], the Kathmandu Declaration (see Appendix A) formalizes participants’ resolve to work together in partnership with other stakeholders to institutionalize sustainable newborn bloodspot screening and to utilize the WHO’s recent position statement to facilitate the implementation of NBS [104]. Because the WHO emphasis is on broader newborn screening, including visible birth defects, NHS, CCHD, and other congenital conditions, and this workshop focused only on bloodspot screening, care was taken to clarify any references to newborn screening with appropriate descriptive terms. Formal reports from the fifth and the sixth workshops in Penang, Malaysia, and Ulaanbaatar, Mongolia, respectively, were never published due to COVID-19 interruptions, and the declarations from these two workshops have been included in Appendix B and Appendix C for completeness.

## 5. Conclusions

Progress in NBS implementation in Asian countries with developing economies is continuing. Over the past 30 years, collaborations across the region and with other interested partners (both public and private) have contributed to successful NBS implementation nationally, with newborn coverage >95%, in a number of Asian countries who have participated in these workshops (China, Korea, Malaysia, the Philippines, Sri Lanka, and Thailand). Significant progress in providing NBS to the newborn population has also been experienced in Vietnam (57% coverage; five conditions—G6PDD, CH, CAH, NHS, and CCHD); Indonesia (28% coverage; CH mandatory, G6PDD and CAH pilots, NHS and CCHD available); and Mongolia (16% coverage; CH, CAH, G6PDD, GALT, and CF; NHS and CCHD available).

There is increasing interest in NBS in Pakistan, and the formation of Pak-IMD-Net has the potential, in combination with efforts ongoing in some hospitals and other venues, to significantly expand NBS coverage nationally. Likewise, a newly established NBS law in one province may serve as a model for others. NBS is still only in its infancy in India, despite pilots dating to the 1980s and extending into the 2020s. NBS in India is progressing on a state-by-state basis, and new government programs focused on improved maternal and child health are beginning to have an impact on NBS. NBS is also still in its initial stages in Bangladesh due, in part, to political unrest and uncertainty. Building on a history of NBS initiated by the BAEC, the emphasis has now moved into the public health arena, and efforts are ongoing to sustain a basic national NBS infrastructure. Other POC screening (hearing, heart, and vision) are also becoming more prevalent. In Laos, international collaboration has demonstrated the effectiveness of NBS and helped to establish a basic NBS system; however, the system is not yet sustainable and is currently inoperable due to funding and supply chain issues. Considerations are ongoing with the MOH to reestablish and continue NBS. In Nepal, building on previous pilots and encouraged by a positive commitment from the Minister of Health, NBS is in Phase I of implementation in Kathmandu with plans for expansion nationally. In Cambodia, pilot testing has demonstrated the need and capability for NBS for CH. Discussions at the ministry level are ongoing, and basic planning has begun. Similarly, NBS in Myanmar is reemerging in value and importance following several years of inactivity related to political uncertainty.

Previous training from IAEA and experiences with various pilot NBS projects have provided a foundation on which to build new NBS programs. While economics contributes to the progress of newborn screening program implementation, it may not be the principal factor. For example, national NBS systems covering over 90% of newborns in Sri Lanka and the Philippines, both considered to be LMICs by the World Bank, have been successfully implemented [2]. In contrast, NBS has not yet reached 50% of newborns in Indonesia (28% coverage) and Mongolia (16% coverage), both UMICs. Similarly, comparisons of IMRs across countries has also not shown commonality at the time NBS was initiated [1,5,6]. Instead, identification, recruitment, and education of local leaders/champions appear to be the more important factors in implementing and sustaining NBS [105,106]. Dedicated NBS champions are demonstrably making a difference in the developing Asian programs. In our workshop, champions from less developed countries obtained valuable knowledge from the experiences of the more developed programs, both regionally and internationally. Their desire to learn was reinforced through the professional and social interactions of the group. The Kathmandu workshop provided up-to-date information and real-life examples of successful NBS implementation in the region, and all attendees have committed to making NBS successful in their home setting.

## Figures and Tables

**Table 1:** Newborns receiving newborn screening in Calmette Hospital, Phnom, Penh, Cambodia.

Year	Screened	CH Cases	G6PDD Cases
2013	7753	2	585
2014	9529	3	638
2015	10,176	5	807
2016	10,381	4	747
2017	12,688	4	978
2018	15,245	7	935
2019	17,632	8	1202
2020	15,035	11	685
2021	7293	6	445
2022	7133	3	606
2023	10,628	6	899
Totals	123,493	59	8527
Abbreviations: CH = congenital hypothyroidism; G6PDD = glucose-6-phosphate dehydrogenase deficiency.			

**Table 2:** Newborn screening statistics for Mongolia—2000–2024.

Date Range	Newborns Screened	Coverage	CH		CAH		GALT		CF	
			Pos	Diag	Pos	Diag	Pos	Diag	Pos	Diag
2000–2002	2897	2.0%	15	2	-	-	-	-	-	-
2012–2014	6849	6.7%	38	2	32	2	-	-	-	-
2015	686	1.6%	2	-	-	-	-	-	-	-
2016–2018	6346	5.3%	11	3	24	2	-	-	-	-
2019–2022	14,766	5.2%	338	7	86	13	-	-	-	-
2023	10,551	15.9%	439	3	17	10	-	-	33	-
2024 (through September)	7836	Incomplete ^a^	169	1	49	-	13	-	15	-
Total (2000–2023)	42,095		1012	18						
Total (2012–2023)	39,198				159	23				
Prevalence			1:2338		1:1452					
^a^ Data for 2024 are not yet complete and have not been included in the prevalence calculations. Abbreviations: CH = congenital hypothyroidism; CAH = 21-hydroxylase deficient congenital adrenal hyperplasia; GALT = transferase deficient galactosemia; CF = cystic fibrosis; Pos = screen positive; Diag = diagnosed case.										

**Table 3:** Preliminary glucose-6-phosphate dehydrogenase deficiency newborn screening statistics for Mongolia—2023–2024.

Year	Newborns Screened	Screen Positive	Percent Screen Positive	Returned for Second Screen	Second Screen Positive	Calculated Prevalence
2023	7952	273	3.4	273	19	1:419
2024 (through September)	6855	278	4.1	278	20	1:343
Total	14,807	551	3.7	551	39	1:380

**Table 4:** Newborn screening statistics for Vietnam—2021–2023.

Date Range	Newborns	Screened	CH		CAH		G6PDD	
			Pos	Diag	Pos	Diag	Pos	Diag
2021	1,152,136	580,378	220	57	53	30	4412	1648
2022	1,046,456	575,615	441	171	171	148	3878	1389
2023	1,159,051	660,201	388	304	119	68	3125	678
Total	3,357,543	1,816,194	1049	532	353	246	11,661	3715
Prevalence	-	-	1:3414		1:7383		1:489	
Abbreviations: CH = congenital hypothyroidism; CAH = 21-hydroxylase deficient congenital adrenal hyperplasia; G6PDD = glucose-6-phosphate dehydrogenase deficiency; Pos = screen positive; Diag = diagnosed case.								

**Table 5:** Selected newborn screening statistics for the Philippines: 1996–2023 *.

1996–2022 *				2014–2023 *			
Condition	Screened	Confirmed	Prevalence	Hb Condition	Screened	Confirmed	Prevalence
G6PDD	17,599,479	282,691	1:62	HbH-Disease ^d^	7,117,133	2932	1:2427
CH	17,678,577	6794	1:2602	HbE-Disease	7,117,133	290	1;24,542
CAH	17,678,577	854	1:20,700	β-Thal ^e^	7,117,133	49	1:145,248
MSUD	13,903,794	221	1:62,903	HbE,β-Thal	7,117,133	27	1:263,598
GAL ^a^	17,687,577	170	1:103,991	HbE,α-Thal	7,117,133	16	1:444,821
HPA ^b^	17,678,577	141	1:125,379	HbS,S-Disease	7,117,133	4	-
GA-1	5,718,974	40	1:142,974	HbS,E-Disease	7,117,133	3	-
3-MCC	5,718,974	29	1:197,206	α-Thal Major	7,117,133	2	-
TYR-1	5,718,974	15	1:381,265	Other ^f^	7,117,133	-	-
Other ^c^	-	-	-				
* Data collection began in 1996 for CH, CAH, GAL, and HPA; in 2000 for G6PDD; in 2012 for MSUD; and in 2014 for MS/MS conditions reported (GA-1, 3-MCC, TYR-1, ASA, IVA, MMA, PA, CIT-I, MAT, and MCD); BIO; and CF; and in 2014 for hemoglobinopathies and thalassemias. Data for hemoglobinopathies and thalassemias include 2023 confirmed cases. Abbreviations: G6PDD = glucose-6-dehydrogenase deficiency; CH = congenital hypothyroidism; CAH = 21-hydroxylase deficient congenital adrenal hyperplasia; MSUD = maple syrup urine disease; GAL = galactosemia; GA-1 = glutaric aciduria type I; 3-MCC = 3-methylcrotonyl-CoA carboxylase deficiency; TYR-1=tyrosinemia type I; Hb = hemoglobin; Thal = thalassemia. ^a^ Includes classical galactosemia (40) and non-classical galactosemia (130). ^b^ HPA = hyperphenylalaninemia Includes classic PKU (23), mild HPA (38), 6-pyruvoyl-tetrahydropterin synthase deficiency (10), and benign HPA (70). ^c^ Other confirmed cases include ASA (8); IVA (5); MMA (5); PA (4); BIO (3); CIT-I (3); MAT (2); MCD (1); CF (1). ^d^ Includes 2917 deletional variants and 15 non-deletional variants. ^e^ Includes β-Thal Major and β-Thal Intermedia—status determined at age 6 months but not yet reported. ^f^ Other hemoglobinopathies detected include HbD disease (1); HbD β-Thal (1).							

## Data Availability

Not applicable.

## Appendix A

**KATHMANDU DECLARATION**
4 September 2024
Kathmandu, Nepal

We, as newborn bloodspot screening program representatives from countries in the Asia Pacific region with less than 50% newborn screening coverage in 2024 (Bangladesh, Cambodia, India, Indonesia, Laos, Mongolia, Myanmar, Nepal, Pakistan, and Vietnam) and expert consultants (the Philippines and USA), have participated in periodic workshops focused on “Consolidating Newborn Bloodspot Screening Efforts in the Asia Pacific Region” to assist in quality program initiation, expansion, and sustainability. Outputs from these workshops have been formalized in declarations by the workshop attendees and include the 2008 Cebu Declaration, the 2010 Manila Declaration, the 2015 Penang Declaration, and the 2017 Ulaanbaatar Declaration. In total, these declarations attest to the importance of collaborative and cooperative networking in the Asia Pacific Region.

All attendees recognize the importance of newborn bloodspot screening as part of newborn care services essential for the good health of every newborn. Further, all attendees fully support Resolution A77/A/CONF./5 of the WHO’s 77th World Health Assembly, entitled “Accelerate progress towards reducing maternal, newborn and child mortality in order to achieve Sustainable Development Goal targets 3.1 and 3.2, 21 June 2024” (available online at: https://apps.who.int/gb/ebwha/pdf_files/WHA77/A77_R5-en.pdf (accessed on 5 September 2024). This resolution “INVITES Member States, in accordance with national context and priorities to consider implementing a universal newborn screening program, including comprehensive birth defect screening, including specific needs and considerations for diagnosis, management and long-term care of children with birth defects.” This includes the implementation of full population newborn screening that focuses on bloodspot screening and other screening appropriate to the specific needs and considerations of the population. Furthermore, improving newborn screening in Lower and Middle-Income Countries is essential in achieving the goals of the 2014 WHO document Every Newborn Action Plan.

As an output of the most recent workshop in Kathmandu, the meeting participants from the twelve countries represented hereby adopt the following statement as part of the Kathmandu Declaration:

Newborn bloodspot screening in an important tool in the prevention of disease, disability, and death in our children and thus, should be a key part of a comprehensive public health system in all of our countries. As a result of the deliberations at the 7th Workshop on Consolidating Newborn Bloodspot Screening Efforts in the Asia Pacific, Kathmandu, Nepal, 2–5 September 2024, the following recommendations are made for prioritization over the next two years.

All countries represented at this workshop shall:

- Continue regionalization and cooperation among countries by sharing expertise, information, and other resources in the implementation of universal newborn bloodspot screening.
- Continue working towards legislation and/or policy development within the Ministry of Health. This legislation/policy will include the provision of necessary support to establish a systematic national newborn bloodspot screening program within the context of a global policy for children’s health. This will provide access and follow-up services to all newborns in these countries. Such services should integrate both public and private healthcare systems.
- Continue developing population studies to determine the incidence of genetic disorders in the region. These data are needed to provide policymakers with a scientific basis for implementing and/or expanding the appropriate newborn bloodspot screening test panel.
- Identify creative funding mechanisms such as inclusion in maternal/newborn/child benefit packages, health/social insurance, or other sources.
- Develop strategies to involve more health facilities in the program.
- Strengthen collaboration within the region.
- Work towards the improvement of the system and operations including:
  ○ Education and capacity-building through training programs that focus on role-specific activities that build interdisciplinary teams. The aim is to increase the number of healthcare professionals, trained specialists, genetic counselors, and nutritionists involved in newborn bloodspot screening;
  ○ Capacity-building through establishing and strengthening newborn bloodspot screening and confirmatory laboratory centers;
  ○ Capacity-building and collaboration for the treatment and long-term care of patients confirmed to have a disorder included in the newborn bloodspot screening panel;
  ○ Increased access to specific treatment and/or medical foods for patients diagnosed through newborn bloodspot screening;
  ○ Establishment of policies providing privacy to patients whose records may be part of newborn bloodspot screening;
  ○ Establishment of external proficiency testing for newborn bloodspot screening laboratories in order to provide optimal quality assurance;
  ○ Periodic monitoring and evaluation of all elements of the newborn bloodspot screening system.
- Increase awareness through development of culturally sensitive advocacy materials, and dissemination through various channels/media.
- Improve access to and utilization of electronic data management systems in support of newborn bloodspot screening.
- Appropriately document satisfactory progress in completing all of the above items.

## Appendix B

**PENANG DECLARATION**
6 December 2015
Penang, Malaysia

In support of the United Nations Convention of the Rights of the child in 1989, all countries recognize that children’s health is a high priority. Considered one of the earliest opportunities of saving the future of children, all countries are encouraged to offer newborn bloodspot screening to all babies at birth. Pursuing this ultimate goal, newborn screening program managers from a group of countries (Bangladesh, Cambodia, China, India, Indonesia, Laos, Mongolia, Nepal, Pakistan, the Philippines, Sri Lanka, Vietnam) in the Asia Pacific Region with less than 50% newborn screening overage in 2008, with expert assistance from the USA, have participated in workshops focused on “Consolidating Newborn Screening Efforts in the Asia Pacific.” The goal of these workshops has been to share newborn screening program experiences in order to assist in program initiation or program expansion. The Cebu Declaration in 2008 and the Manila Declaration in 2010 were initiated by workshop attendees to formally recognize the need for collaborative and cooperative networking in order to facilitate the development of sustainable newborn screening systems. This Penang Declaration is put forth to further emphasize this need and to document the need for continuing workshops of this nature in the future.

As an output of the most recent workshop in Penang, Malaysia, the meeting participants from these 11 countries in attendance (Cambodia, China, India, Indonesia, Mongolia, Nepal, Pakistan, the Philippines, Sri Lanka, Vietnam, and USA) hereby adopt the following Penang Declaration:

Newborn screening is an important tool in the prevention of disease and disability in our children and this should be a key part of a comprehensive public health system in all of our countries. As a result of deliberations at the 5th Workshop on Consolidating Newborn Screening Efforts in the Asia Pacific, Penang, Malaysia, 5–6 December 2015, the following recommendations are made for prioritization over the next two years.

All countries represented at this workshop shall:

- Continue regionalization and cooperation among countries by sharing expertise, information and other resources.
- Continue working towards legislation and/or policy development within the Ministry of Health. This legislation/policy will include the provision of necessary support to establish as systematic national newborn screening program within the context of a global policy for children’s health. This will provide access fand follow-up services to all newborns in these countries. Such services should integrate public and private healthcare systems.
- Continue developing population studies to determine the incidence of genetic disorders in the region. These data are needed to provide policymakers with a scientific basis for implementing and/or expanding the appropriate newborn screening testing panel.
- Develop strategies to involve more health facilities in the program and to strengthen collaboration within the region.
- Work towards the improvement of the system and operations, including:
  ○ Capability building through training programs that focus on role-specific activities that build interdisciplinary teams. The aim is to increase the number of trained specialists, genetic counselors, and nutritionists involved in the program;
  ○ Capacity building through establishment and strengthening of confirmatory laboratory centers;
  ○ Increased access to treatment and medical foods for patients;
  ○ Establishment of policies on the retention and use of the residual dried blood spots remaining after completion of screening;
  ○ Establishment of policies providing privacy to patients whose records may be a part of newborn screening;
  ○ Establishment of external proficiency testing for newborn screening laboratories in order to provide optimal quality assurance.
- Increase awareness through development of culturally sensitive advocacy materials and dissemination through various channels/media.
- Identify creative funding mechanisms, such as inclusion in maternal/child health packages and health/social insurance.

## Appendix C

**ULAANBAATAR DECLARATION**
28 August 2017
Ulaanbaatar, Mongolia

We, the participants of the 6th Workshop on Consolidating Newborn Screening Efforts in the Asia Pacific (composed of representatives from Bangladesh, Cambodia, China, India, Indonesia, Malaysia, Mongolia, Nepal, Pakistan, the Philippines, Sri Lanka, and Vietnam), with expert assistance from the United States, held on 26–28 August 2017, reaffirm our commitment to the principles and provisions listed in the Cebu, Manila, and Penang Declarations (http://www.newbornscreening.ph).

In support of the United Nations Convention of the Rights of the child in 1989, all countries recognize that children’s health is a high priority. Considered one of the earliest opportunities of saving the future of children, all countries are encouraged to offer newborn bloodspot screening to all babies shortly after birth. Pursuing this ultimate goal, we as newborn screening program managers of countries in the Asia Pacific Region with less than 50% newborn screening coverage in 2008, have participated in periodic workshops focused on “Consolidating Newborn Screening Efforts in the Asia Pacific”. The goal of these workshops has been to share newborn screening program experiences with each other with the aid of input from a recognized international newborn screening expert in order to assist in program initiation, expansion sustainability, and quality.

Since 2008, China, the Philippines, and Sri Lanka have achieved 93%, 83%, and 82% newborn screening coverage, respectively. The remaining countries have increased their newborn screening coverage but continue to be below the 50% threshold. Among the group, the newborn screening programs in China, Philippines, Sri Lanka and Vietnam have formally connected with their national public health ministries.

In addition, although Malaysia has accomplished 98% newborn screening coverage, sustainable expansion to other screenable conditions has been limited by the sample currently collected, which is cord blood. In order to obtain information about possible screening benefits that might be achieved by using a dried bloodspot specimen, a country representative attended the current workshop as an observer.

The 2008 Cebu Declaration, 2010 Manila Declaration, and 2015 Penang Declaration were previously initiated by workshop attendees to formally recognize the need for collaborative and cooperative networking for this purpose. This 2017 Ulaanbaatar Declaration is put forth to further emphasize these needs and to document the value of these workshops in the past and to reaffirm their continuing importance.

As an output of the most recent workshop in Ulaanbaatar, the meeting participants from these 13 countries hereby adopt the following Ulaanbaatar Declaration:

Newborn screening is an important tool in the prevention of disease disability and death in our children and thus should be a key part of a comprehensive public health system in all of our countries. As a result of deliberations at the Ulaanbaatar Workshop on Consolidating Newborn Screening Efforts, 26–28 August 2017, the following recommendations are made for prioritization over the next two years.

All countries represented at this workshop shall:

- Continue regionalization and cooperation among countries by sharing expertise, information, and other resources.
- Continue working towards legislation and/or policy development within the Ministry of Health. This legislation/policy will include the provision of necessary support to establish a systematic national newborn screening program within the context of a global policy for children’s health. This will provide access and follow-up services to all newborns in these countries. Such services should integrate both public and private healthcare systems.
- Continue developing population studies to determine the incidence of genetic disorders in the region. These data are needed to provide policymakers with a scientific basis for implementing and/or expanding the appropriate newborn screening testing panel.
- Develop strategies to involve more health facilities in the program and to strengthen collaboration within the region.
  ○ Capability building through training programs that focus on role-specific activities that build interdisciplinary teams. The aim is to increase the number of trainee specialists, genetic counselors, and nutritionists involve in the program.
  ○ Capacity building through establishment and strengthening of confirmatory laboratory centers.
  ○ Increased access to treatment and medical foods for patients.
  ○ Establishment of policies on the retention and use of the residual dried blood spots remaining after completion of screening.
  ○ Establishment of policies providing privacy to patients whose records may be a part of newborn screening.
  ○ Establishment of external proficiency testing for newborn screening laboratories in order to provide optimal quality assurance.
- Increase awareness through development of culturally sensitive advocacy materials and dissemination through various channels/media.
- Identify creative funding mechanisms such as inclusion in maternal/child health packages and health/social insurance.
